# Assessment of *Pseudomonas aeruginosa N* ^5^,*N* ^10^-Methylenetetrahydrofolate Dehydrogenase - Cyclohydrolase as a Potential Antibacterial Drug Target

**DOI:** 10.1371/journal.pone.0035973

**Published:** 2012-04-25

**Authors:** Thomas C. Eadsforth, Mary Gardiner, Fernando V. Maluf, Stuart McElroy, Daniel James, Julie Frearson, David Gray, William N. Hunter

**Affiliations:** Division of Biological Chemistry and Drug Discovery, College of Life Sciences, University of Dundee, Dundee, United Kingdom; University of South Florida College of Medicine, United States of America

## Abstract

The bifunctional enzyme methylenetetrahydrofolate dehydrogenase – cyclohydrolase (FolD) is identified as a potential drug target in Gram-negative bacteria, in particular the troublesome *Pseudomonas aeruginosa*. In order to provide a comprehensive and realistic assessment of the potential of this target for drug discovery we generated a highly efficient recombinant protein production system and purification protocol, characterized the enzyme, carried out screening of two commercial compound libraries by differential scanning fluorimetry, developed a high-throughput enzyme assay and prosecuted a screening campaign against almost 80,000 compounds. The crystal structure of *P. aeruginosa* FolD was determined at 2.2 Å resolution and provided a template for an assessment of druggability and for modelling of ligand complexes as well as for comparisons with the human enzyme. New FolD inhibitors were identified and characterized but the weak levels of enzyme inhibition suggest that these compounds are not optimal starting points for future development. Furthermore, the close similarity of the bacterial and human enzyme structures suggest that selective inhibition might be difficult to attain. In conclusion, although the preliminary biological data indicates that FolD represents a valuable target for the development of new antibacterial drugs, indeed spurred us to investigate it, our screening results and structural data suggest that this would be a difficult enzyme to target with respect to developing the appropriate lead molecules required to underpin a serious drug discovery effort.

## Introduction

The Gram-negative *Pseudomonas aeruginosa* is a serious nosocomial pathogen, accounting for a significant level of hospital-acquired infections and is particularly troublesome for burn victims, immunocompromised and cystic fibrosis patients [1], [2]. Two major factors contribute to this health problem. Firstly, the bacterium can survive moist, low nutrient conditions and therefore persist in the clinical environment. Secondly, numerous drug resistant strains of *P. aeruginosa*, employing the common mechanisms of resistance such as modification of the target, active efflux and/or decreased uptake of drugs, have emerged [3]–[6]. The need for novel antibiotics to tackle, in particular Gram-negative bacteria such as *P. aeruginosa*, and drug resistant bacteria in general, has been well publicized along with the practical difficulties associated with antibacterial drug development [7], [8]. However, there are now genome sequences available for important pathogens and increasing knowledge of the mechanism of action of existing drugs. Data are available on which genes encode essential activities and we have an improved understanding of what types of molecules are likely to provide either the drug targets or appropriate lead compounds [9], [10]. It is therefore appropriate and timely to identify and carefully assess potential targets that might provide a foundation for the future of antimicrobial research.

**Figure 1:**
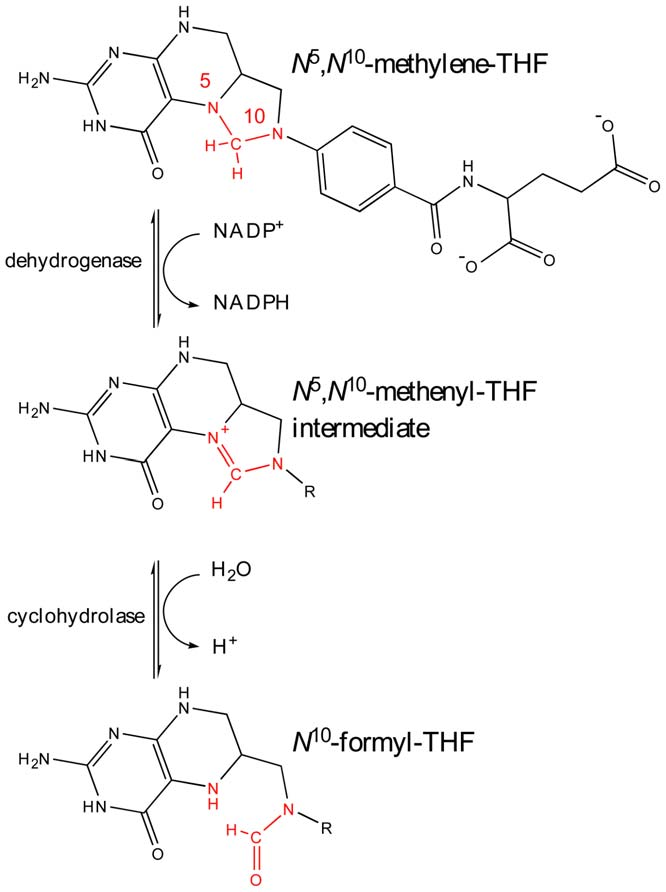
The reaction catalyzed by FolD. *N* ^5^,*N* ^10^-methylene-THF is converted to *N* ^5^,*N* ^10^-methenyl-THF and subsequently *N* ^10^-formyl-THF in a two-step reaction, initially in an NADP^+^ or NAD^+^ dependant oxidization to by *N* ^5^,*N* ^10^-methylenetetrahydrofolate dehydrogenase [DH, EC:1.5.1.5] and subsequent hydrolysis by *N* ^5^,*N* ^10^-methenyltetrahydrofolate cyclohydrolase [CH, EC:3.5.4.9].

One area of bacterial metabolism that has been successfully targeted by antibacterial drugs is the folate biosynthetic pathway. The enzymes that synthesize, link and modify tetrahydrofolate (THF) maintain the cellular levels of important cofactors such as methenyl-, methylene-, formyl- and unsubstituted THF. These compounds are essential for the synthesis of thymidine, purines, glycine, methionine, initiator fMet-tRNA and also in the metabolism of histidine and serine [11]. Higher eukaryotes obtain these cofactors mainly through diet, whilst bacteria are able to synthesize these valuable nutrients. Folate biosynthesis depends on enzymes such as dihydropteroate synthase and dihydrofolate reductase and inhibitors of these enzymes are used to treat microbial infections [12]–[14]. Inspection of folate metabolism in *P. aeruginosa* drew our attention to the bifunctional enzyme methylenetetrahydrofolate dehydrogenase - cyclohydrolase. This enzyme converts *N* ^5^,*N* ^10^-methylene-THF to *N* ^10^-formyl-THF in a two step reaction, initially in an NADP^+^ or NAD^+^ dependant oxidization to *N* ^5^,*N* ^10^-methenyl-THF by *N* ^5^,*N* ^10^-methylenetetrahydrofolate dehydrogenase [DH, EC:1.5.1.5] and subsequent hydrolysis to *N* ^10^-formyl-THF by *N* ^5^,*N* ^10^-methenyltetrahydrofolate cyclohydrolase [CH, EC:3.5.4.9] (Figure 1). The gene encoding this cytosolic enzyme, *folD*, has been shown by knock-out studies to be essential in the Gram-positive *Bacillus subtilis*, as well as the Gram-negative *Escherichia coli*, *Francisella novicida*, *Acinetobacter baylyi*, and in *P. aeruginosa* itself [15]–[19].

We first considered a diverse set of criteria that have been established as key areas with respect to target assessment for early stage antimicrobial drug discovery [20]. The criteria include genetic and chemical validation of the target, druggability, the feasibility of an assay, the potential for toxicity and also for drug resistance, and the availability of accurate structure information to guide the development of structure-activity relationships. Our objective was to elucidate the potential of *P. aeruginosa* FolD (*Pa*FolD) as a point of therapeutic intervention and to identify what further information was necessary to provide a comprehensive assessment. As mentioned, the *folD* gene has been shown to be essential in *P. aeruginosa* providing genetic validation of the target [19].

Potent inhibitors of FolD are known, including substrate analogues, and these provide standard compounds and chemical information concerning modes of inhibition [21]–[23]. These inhibitors display biological activity as antiproliferative agents of mammalian cells but there is no published evidence of antibacterial properties [21]. In mammals it appears that the dehydrogenase - cyclohydrolase activity is necessary for early development but that adult tissues are less dependent. A potential mechanism for resistance that might circumvent FolD inhibition is up regulation of *N* ^10^-formyl-THF biosynthesis as observed in the protozoan *Leishmania major* [24]. An enzyme assay for FolD is available and appeared suitable for conversion into a high-throughput screening (HTS) format [25]. Active recombinant material has been prepared and structural data are available for several FolDs including the human and *E. coli* enzymes, *Hs*DHCH and *Ec*FolD respectively, though not for *Pa*FolD itself [23], [26], [27], [28].

We decided that a thorough assessment of *Pa*FolD as a therapeutic target required structural and compound screening data and that this would also support the search for new inhibitors that might represent useful lead compounds. We describe the preparation of an efficient recombinant protein production system, protocols for purification and crystallization. The crystal structure has been determined allowing for a druggability analysis of the active site and detailed comparisons with other FolD structures including that of the human enzyme [26]. Screens of a fragment library and a collection of bioactive molecules were carried out using differential scanning fluorimetry (DSF). An appropriate enzyme assay was developed and then applied in an HTS screen. Enzyme assay and surface plasmon resonance (SPR) measurements were subsequently used to evaluate and characterize the hits. The data package allows us to assess the tractability of FolD for antibacterial drug discovery.

## Materials and Methods

### Recombinant source of *Pa*FolD

The *P. aeruginosa folD* gene, encoding the bifunctional *N* ^5^,*N* ^10^-methylene tetrahydrofolate dehydrogenase/*N* ^5^,*N* ^10^-methenyl tetrahydrofolate cyclohydrolase, was identified in Swissprot (http://expasy.org/sprot/ accession number Q9I2U6 and the genome website (http://www.pseudomonas.com/). The gene (locus tag: PA1796) was amplified from genomic DNA (American Type Culture Collection 47085, strain PAO1) with the primers carrying *Nde*I and *Xho1* restriction sites (bold), respectively: 5′- **CAT-ATG**-ACC-GCA-CAA-CTG-ATC-3′, 5′- **CTC-GAG**-TCA-GTC-GTG-CAG-G-3′. The PCR product was ligated into pCR-BluntII-TOPO vector using the Zero Blunt TOPO PCR cloning kit (Invitrogen). The gene was excised and ligated into a modified pET15b vector (Novagen) containing a Tobacco Etch Virus (TEV) protease recognition sequence in place of thrombin (pET15bTEV). This results in a product carrying an N-terminal hexa-histidine tag (His-tag), which is cleavable with TEV protease. The recombinant plasmid was amplified in XL-1 blue *E. coli*, and the gene sequence verified, before being transformed into *E. coli* BL21 (DE3) for protein production.

### Purification of *Pa*FolD

*E. coli* carrying the *P. aeruginosa FolD*-pET15BTEV plasmid were cultured at 37°C with shaking at 200 rev min^−1^ in auto-induction media supplemented with 50 mg L^−1^ carbenicillin for approximately three hours until an OD_600_ of 0.8 was reached. The temperature was then reduced to 21°C followed by expression for 22 hours. Cells were harvested by centrifugation (30 min, 3,500 *g*, 4°C) prior to re-suspension in lysis buffer (buffer A: 50 mM Tris-HCl pH 7.5, 250 mM NaCl and 20 mM imidazole) containing DNAse I (100 µg) and an EDTA-free protease-inhibitor cocktail tablet (Roche). Cells were lysed using a French press at 16,000 psi. Insoluble debris was separated by centrifugation (50,000 *g*, 30 min, 4°C) and the soluble fraction was filtered and loaded onto a HisTrap HP 5 mL column (GE Healthcare) previously charged with Ni^2+^. The His-tagged protein was eluted with a 0–1 M imidazole gradient in the same buffer. Histidine-tagged TEV protease (1 mg per 20 mg FolD) was added and the mixture dialyzed against 50 mM Tris-HCl pH 7.5 and 250 mM NaCl for three hours. Passage through a His-Trap column separated *Pa*FolD from TEV protease, the cleaved His-tag peptide and uncleaved His-tag *Pa*FolD. The sample of *Pa*FolD was then applied to a Superdex 200, 26/60 column (GE Healthcare), pre-equilibrated in buffer A. The protein eluted as a dimer with a molecular mass of approximately 60 kDa. *Pa*FolD was then concentrated (10 kDa MWCO Amicon Ultra devices, Millipore) to 15 mg mL^−1^. Protein concentration was determined spectrophotometrically using a theoretical extinction coefficient of 6,150 mol L^−1^ cm^−1^ at 280 nm calculated using ProtParam [29]. The high level of protein purity was confirmed by SDS-PAGE and matrix-assisted laser desorption ionization-time-of-flight mass spectrometry.

### Preparation of biotinylated *Pa*FolD for SPR

For analysis of compounds using SPR it was necessary to produce an expression system that provided a protein carrying a biotinylation acceptor peptide (BAP-tag). This BAP-tag peptide has the sequence 5′ - GLNDIFEAQKIEWHE - 3′ and is specifically biotinylated by the *E. coli* biotin holoenzyme synthetase, BirA. The modified *Pa*FolD was then purified as before. Subsequently 30 µM of BAP-tagged *Pa*FolD was incubated at 37°C overnight in buffer C (10 mM Tris-HCl pH 7.5, 200 mM KCl, 5 mM MgCl_2_, 100 µM d-biotin) containing 500 µM ATP and 1 µM BirA. The sample was passed through a HisTrap HP column equilibrated with buffer A to remove BirA. The flow through was collected and concentrated (Amicon 10 kDa MWCO spin column) to remove free biotin. The incorporation of biotin was monitored by MALDI-TOF analysis performed at the University of Dundee ‘Fingerprints’ Proteomics Facility using an Applied Biosystems Voyager DE-STR spectrometer. The biotinylated protein was flash frozen at 10 mg mL^−1^ in 50 mM Tris-HCl, 250 mM NaCl, 15% glycerol, pH 7.5 and stored at −80°C until required.

### Surface Plasmon Resonance (SPR)

A Biacore T100 instrument (GE Healthcare) was used for all SPR experiments. Biotinylated *Pa*FolD was diluted 50-fold into a running buffer containing 50 mM Tris-HCl, pH 7.5, 150 mM NaCl, 5 mM MgCl_2_, 1 mM DTT, 0.05% Tween 20, 1 mM NADP^+^ and 1% DMSO and injected over a streptavidin chip (GE Healthcare) at a flow rate of 10 µL min^−1^ for 10 min to obtain a density of ∼4,000 response units. Compounds DDD55519 and DDD61461 were injected in duplicates at three-fold concentration series 136 nM–11 µM at a flow rate 30 µL min^−1^. Association was measured for 1 min and dissociation for 20 min. Compounds DDD32388 and DDD58331 were injected at three-fold concentration series of between 1.23 µM–100 µM at a flow rate 30 µL min^−1^. Association was measured for 1 min and dissociation 2 min. Data were referenced from a blank streptavidin surface and blank injections of buffer. Processing was carried out using Scrubber 2 software (BioLogic Software, Australia).

### Fluorescence-based screening by differential scanning fluorimetry (DSF)

DSF was used to screen different buffers and concentrations of cofactor to identify conditions under which the protein displayed optimum thermal stability [30], [31]. It was reasoned that such conditions would favour crystallization. In addition two compound libraries, the Maybridge fragment set and the Prestwick collection of biologically active molecules, were screened after first assessing the suitability of the method for *Pa*FolD, the optimum buffer and cofactor conditions and the lowest concentration of protein that generated a strong signal as previously described for our laboratory [32]. An Mx3005p RT PCR system (Stratagene) was used to monitor protein unfolding by the increase in fluorescence of SYPRO Orange dye (Invitrogen). Briefly, the enzyme was tested against 1000 compounds from the Maybridge Rule of Three (Ro3) fragment library and 1120 compounds from the Prestwick Chemical Library. Assays were carried out in 40 µL volumes with *Pa*FolD at 4 µM, supplemented with 4 mM NADP^+^ in 50 mM Tris-HCl, 250 mM NaCl, pH 7.5 in 96 well RT PCR plates (Abgene). The compounds, 1 µL dissolved in DMSO, were incubated with the protein solution for 5 minutes prior to 71 cycles of 1°C temperature increments starting at 25°C. After each 1°C increase the sample was excited at 492 nm and fluorescence emissions recorded at 610 nm. The melting temperatures were plotted against a reference control sample of DMSO only and each plate contained two known inhibitors of FolD as quality control measures (LY354899 and LY374571). Compound concentrations varied between 1 mM for the Maybridge library and between 2 mM and 8 mM for the Prestwick library (compounds at 1 mg mL^−1^) with a requirement to limit the concentration of DMSO to <2.5% in the final mixture.

### Crystallization and data collection

Sitting drop vapour diffusion crystallization trials were carried out using a Phoenix Liquid Handling System (Art Robins Instruments/Rigaku) and the JCSG+ MPD, PEG and Classics screens (Hampton Research). The trials used drops, assembled from 100 nL of protein solution and an equivalent volume of reservoir, equilibrated against a 70 µL reservoir at 20°C. Crystals were observed after three days in conditions with a reservoir of 25% PEG 3350 and 0.2 M magnesium formate. Optimization by hanging drop (2 µL volume) vapour diffusion gave crystals with approximate dimensions 0.3×0.2×0.1 mm^3^. Single crystals were transferred to a cryo-solution containing the original reservoir solution supplemented with 40% glycerol prior to flash freezing at −173°C. Crystals were first characterized in-house with a Micromax-007 rotating anode generator and R-AXISIV^++^ dual image plate detector (Rigaku), prior to storage in liquid nitrogen. X-ray diffraction data were then collected at beam line ID29 at the European Synchrotron Radiation Facility using a wavelength of 0.9814 Å. Integration and scaling of data were carried out using MOSFLM and SCALA [33], [34]. The crystals are monoclinic with space group *P*2_1_ and unit cell dimensions of *a* = 61.42 Å, *b* = 82.38 Å, *c* = 109.90 Å, *β* = 94.7°. The molecular weight of a subunit is 30.7 kDa, and the asymmetric unit consists of four subunits with a V_M_ value of 2.5 Å^3^ Da^−1^ and solvent content of approximately 50%.

### Structure solution and refinement

The structure was solved by molecular replacement using a monomer from the *Ec*FolD structure (sequence identity of 67%, PDB code 1BOA) as the search model [27]. The side chains of the search model were removed and the rotation and translation functions (PHASER) positioned four molecules in the asymmetric unit. Inspection using the graphic software COOT showed that two homodimers, consistent with the gel filtration results, formed the asymmetric unit [35], [36]. Rigid-body refinement was carried out in REFMAC5 [37]. Side chains were added to the model based on inspection of electron and difference density maps, followed by iterative rounds of restrained refinement, model building/manipulation and addition of solvent molecules. Translation/Libration/Screw analysis was applied in the latter stages of the refinement [38]. Model quality was checked using MolProbity [39]. Structure superpositions were calculated using LSQKAB and figures were prepared using PyMOL [40], [41].

### FolD enzyme assay development

Enzyme activity was assayed by measuring the absorbance at 350 nm of the intermediate product in the reaction, *N* ^5^,*N* ^10^-methenyl-THF, following acidification of the reaction and consequent reconversion of the final product formyl-THF to the intermediate [25]. Following optimization of the assay buffer and determination of the enzyme linearity, assays were carried out at room temperature in a 50 µL reaction volume containing 25 mM bicine, pH 7.9, 1 mM dithiothreitol, 0.05% CHAPS, 0.2 mg mL^−1^ BSA, 1 nM recombinant FolD, 250 mM NADP^+^ and 35 µM *N* ^5^,*N* ^10^-methylene-THF. Michaelis constants for the two substrates (NADP^+^ and *N* ^5^,*N* ^10^-methylene-THF) were determined in an end-point assay, using these buffer and enzyme conditions.

### FolD hit identification

The HTS was performed with a compound collection of 79,029 diverse structures based around 4000 chemical scaffolds. All compounds were dissolved in 100% DMSO to a concentration of 3 mM. Single point inhibition assays were carried out at room temperature in clear, flat bottom, polystyrene, 384-well plates (Matrix). Each assay was performed in a 50 µL reaction volume as described above. A standard compound (0.5 µL in DMSO) was transferred to all assay plates using a Cartesian Hummingbird (Genomics Solutions) before 25 µL of a reaction mix, containing all assay components except NADP^+^ and *N* ^5^,*N* ^10^-methylene-THF, was added to assay plates using a Thermo Scientific Well-Mate (Matrix). The reaction was initiated and stopped with the additions of 25 µL of substrate and 50 µL of 1 M HCl, respectively, again using a Well-Mate. The FolD assay was run at room temperature for 20 min and the signal was allowed to develop for 10 min before the absorbance of each well was read at 350 nm using an EnVision multilabel plate reader (PerkinElmer Life Sciences). ActivityBase (ID Business Solutions) was used for data processing and analysis.

### FolD inhibitor studies

A high hit rate was noted in the primary screen therefore it was decided to focus on compounds with percentage inhibition values of 80% or greater for follow up potency testing. Compounds of interest were cherry picked from the original library plates using a series of 10-point inhibitor curves (consisting of half-log serial dilutions of compound in DMSO) and prepared in 384-well plates using a JANUS workstation (PerkinElmer Life Sciences). Each compound plate produced 10-point inhibitor curves for 30 test compounds and two curves for LY374571, the standard compound in this screen (see following paragraph for inhibitor details). Following preparation of the inhibitor curves, assays were carried out as described above. ActivityBase was again used for data processing and analysis. All IC_50_ curve fitting was undertaken within ActivityBase XE utilizing the underlying ‘MATH IQ’ engine of XLfit version 5.1.0.0. A four-parameter logistic dose-response curve was utilized for compound potency determination.

### Compounds for HTS

The substrate, *N* ^5^,*N* ^10^-methylene-THF, was purchased from Schirks laboratories, whilst inhibitors LY354899 and LY374571 were synthesised according to previously reported methods [21], [23] and analyzed by NMR, mass spectrometry and high performance liquid chromatography. All chemicals utilized were of analytical grade.

### Molecular docking

Compounds identified by HTS were positioned into the active site of the “open” form of *Pa*FolD using the molecular graphics program COOT. The position of LY354899 bound to the crystal structure of *Hs*FolD, following least-squares superposition of the two enzyme structures, provided a suitable template to guide this modelling. The active site was prepared for docking of the ligand using ICM Pro (Molsoft) with the centre of the ligand-binding site defined by a cavity that contained the residues within 5 Å of LY354899. The top ten docking poses, as scored by ICM Pro, were subsequently inspected.

### Disc diffusion sensitivity testing

Compounds identified by HTS were used in a disc diffusion sensitivity test against *P. aeruginosa*. Briefly a single colony of *P. aeruginosa* PAO1 ATCC 15692 was used to inoculate a 2 mL volume of LB media prior to overnight growth at 37°C. The bacteria were then diluted 1∶100 fold prior to 100 µL volumes plated onto Iso-Sensitest agar plates and dried in air for 5 minutes. Eight 3 mm discs were impregnated with 5 µl of compound, dissolved in DMSO, prior to loading onto each plate. Two controls were used per plate, one a 100% DMSO stock, the other a 10 µg stock of gentamycin. Six compound dilutions were tested per plate, ranging from approximately 115 µg to 4.5 ng. Three compounds with known antifolate activity were tested, namely methotrexate, LY354899 and LY374571 in addition to the three singletons, DDD32388, DDD55519 and DDD61461, and two of the biaryl sulphonamide series that had been identified. Plates were incubated at 37°C, and zones of inhibition measured after 16 and 48 hours.

### Accession number

Coordinates and structure factor data have been deposited with the PDB, code 4A5O.

## Results and Discussion

### Structural analysis

**Figure 2:**
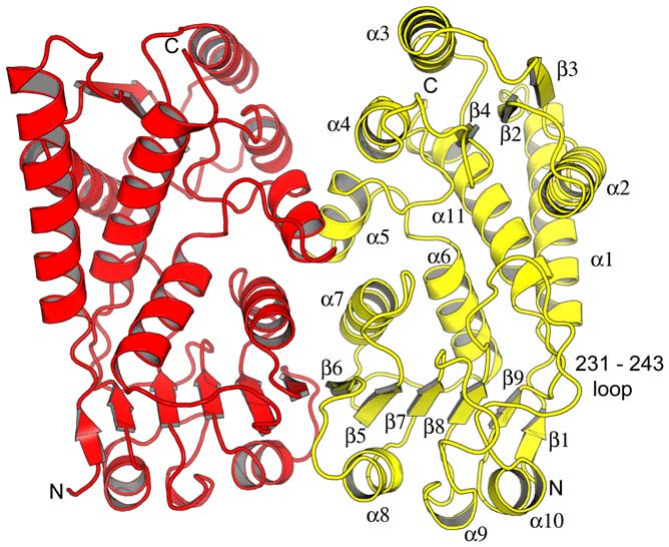
Structure of *Pa*FolD. Cartoon representation of a homodimer of *Pa*FolD with secondary structure labeled. The interface occurs between α5, α7 and β6 of partner subunits.

An efficient supply of recombinant material, yielding over 30 mg of enzyme per litre of bacterial culture, and an efficient purification protocol were established. This provided a source of enzyme for structural studies and a HTS campaign. Ordered crystals were obtained and the structure of *Pa*FolD, with four molecules in the asymmetric unit, was solved using a monomer of *Ec*FolD as the search model for molecular replacement calculations. *Pa*FolD and *Ec*FolD share 67% sequence identity [27]. The structure was subsequently refined to 2.2 Å resolution. Crystallographic statistics are given in Table 1. The four monomers, labelled A–D, are arranged as two homodimers, consistent with the observation of dimeric species in size exclusion gel chromatography and with previously determined FolD structures [26]–[28]. We only detail the A∶B dimer (Figure 2) since a least-squares fit of 280 Cα atoms with an RMSD of 0.4 Å indicate that the subunits are similar, compared to between 0.4 and 0.8 for the other subunit and the electron density is better defined for this pair compared to the other homodimer. This is reflected in a slightly lower average thermal or B-factor value of 44 Å^2^ for the A∶B dimer compared to 73 Å^2^ for the C∶D combination. In addition a loop, residues 233–241, is disordered in chain D, whilst it is ordered in the other subunits.

**Table 1:** Crystallographic statistics.

Spacegroup	*P*2_1_
Unit cell parameters	61.57 Å, 82.43 Å, 109.07 Å, 90°, 94.7°, 90°
Resolution range (Å)**^A^**	40 - 2.2 (2.32 - 2.2)
Wavelength (Å)	0.9814
Number of measurements	200243 (29494)
Number of unique reflections	55263 (8034)
Multiplicity	9.5 (2.6)
Completeness (%)	99.9 (100)
Mean I/σI	3.6 (3.7)
Wilson *B* (Å^2^)	41.6
R_merge_ **^B^**	0.073(0.477)
R_work_ **^C^**	0.23
R_free_ **^D^**	0.277
RMSD bonds (Å)	0.0073
RMSD angles (°)	1.052
Ramachandran (%)**^E^**	
Favoured	96.9
Allowed	2.9
Outliers	0.2
Protein residues	1123
Protein atoms total	8501
Overall *B* (Å^2^)	42.9/44.5/73.1/72.0
Waters	135
Overall *B* (Å^2^)	39.8
PEG/Glycerol	1/1
Overall *B* (Å^2^)	40.8/62.9
Dual occupancy residues	53A, 99A, 133A, 235A, 133B
Missing residues	1A, 1C, 1D, 233-241D, 284D
Low occupancy (Chain A)	18, 21, 59, 85, 191
Low occupancy (Chain B)	18, 21, 27, 56, 59, 64, 194, 212, 223
Low occupancy (Chain C)	2, 9, 14, 15, 21, 22, 24, 27, 29, 31, 33, 43, 48, 51, 59, 63, 64, 68, 70, 73, 78, 79, 80, 137, 149, 194, 240, 271
Low occupancy (Chain D)	9, 18, 22, 23, 25, 27, 29, 31, 33, 54, 56, 59, 61, 63, 64, 118, 138, 194, 212, 217, 223, 247, 251, 252, 275, 282

(**A**) Values in parentheses refer to the highest resolution bin of 2.32 - 2.2 Å (**B**). *R_merge_* = Σ*hΣi*||(*h,i*)−<*I*(*h*)>Σ*hΣi* I(*h,i*) (**C**) *R_work_* = Σ*hkl*||*F_o_*|−|*F_c_*||/Σ*|F_o_|*, where *F_o_* is the observed structure factor and *F_c_* the calculated structure factor (**D**). *R_free_* is the same as *R_work_* except calculated using 5% of the data that are not included in any refinement calculations (**E**) Ramachandran analysis from MOLPROBITY [44].

**Figure 3:**
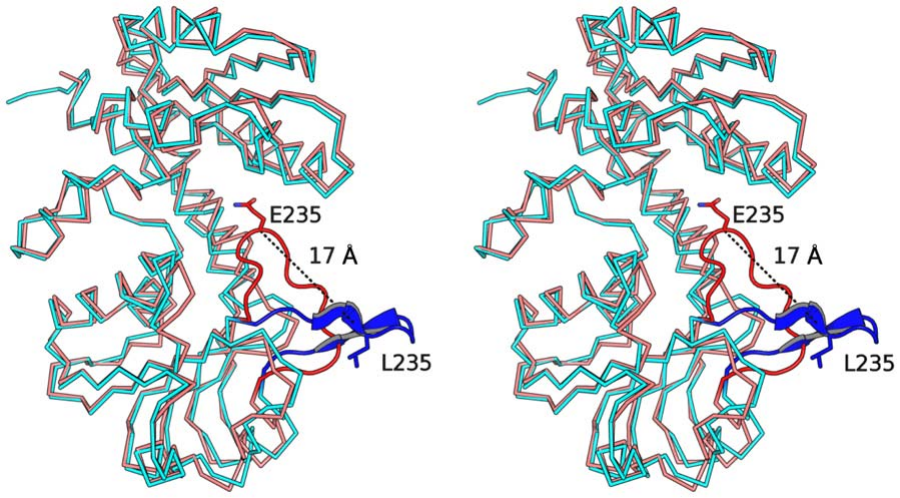
Different loop conformations at the active site. Superposition of a subunit of *E.coli* FolD (PDB code: 1B0A black) against *Pa*FolD. A loop in the *Pa*FolD structure (red residues 231–243) adopts a different orientation compared to the *Ec*FolD structure (blue) with equivalent residues (Gln235 *Pa* and Leu235 *Ec*) shifting by as much as 16.7 Å and an angle of nearly 60°. In the orientation seen for the *Pa*FolD structure, the loop sits over the active site.

The *Pa*FolD subunit consists of 284 residues arranged as a two domain structure, each with an α/β fold, connected by two long helices. The dimer interface is created by interactions involving residues on α5, α7 and β6 (Figure 2). Detailed comparison of the *Pa*FolD structure with other FolD structures, in particular *Ec*FolD indicates that a loop linking β8 and α10 (residues 231–243) is significantly different and occludes the active site. A least-square fit of 280 Cα positions gives an overall RMSD of 2.1 Å. However, for 13 residues on the loop the RMSD averages to 7.5 Å, with the largest deviation of 16.4 Å occurring for residue 235 (Figure 3). Omission of this loop for subsequent least-squared fits reduces the RMSD to 1 Å over 269 Cα positions. Since the recombinant *Pa*FolD retains catalytic activity we assign the loop conformation as an artefact of crystallization but which nonetheless indicates a flexibility likely relevant to the enzyme activity. Our attempts to soak inhibitors into the *Pa*FolD crystals resulted in either the crystals breaking up or the structure revealing the same loop configuration with no density to suggest ligand binding (data not shown). Attempts to grow crystals of the enzyme in a different form or by co-crystallization in the presence of ligands and inhibitors failed to produce diffraction quality crystals. It was therefore necessary to model an open form of *Pa*FolD. Using *Ec*FolD and *Hs*FolD structures as a guide, the active site loop was remodelled to open up that active site. In addition, other loops (94–113, 37–64, 165–172, 207–226, 230–268) were manipulated to convert the *Pa*FolD structure to be more similar to FolD/DHCH ligand structures previously published. It was this model of *Pa*FolD, with a more open active site that was used for molecular docking studies.

A druggable protein target would present a well-defined and ordered cavity, typically with pronounced hydrophobic character, able to bind small bioavailable molecules with high affinity [42]. The active site of dihydrofolate reductase (DHFR), an archetypal target for treatment of cancer and microbial infections, would be described as druggable. The volume of the DHFR active site is estimated as 380 Å^3^ when calculated from Protein Data Bank (PDB) codes 2X9G and 3CL9 [43]. Based on crystal structures of FolD, including that reported here, we estimate the active site to occupy a volume of approximately 430 Å^3^. This is only a modest increase on DHFR and with some parts of the active site displaying hydrophobic character (discussed shortly) then the active site of FolD would be considered well suited to bind drug-like molecules. Indeed such inhibitors of human FolD have been identified supporting the conclusion that the active site is indeed druggable [23]. These inhibitors were primarily developed for their potential as anticancer agents since the folate pathway produces essential co-factors for cell division.

### Comparison of *Pa*FolD and *Hs*FolD

**Figure 4:**
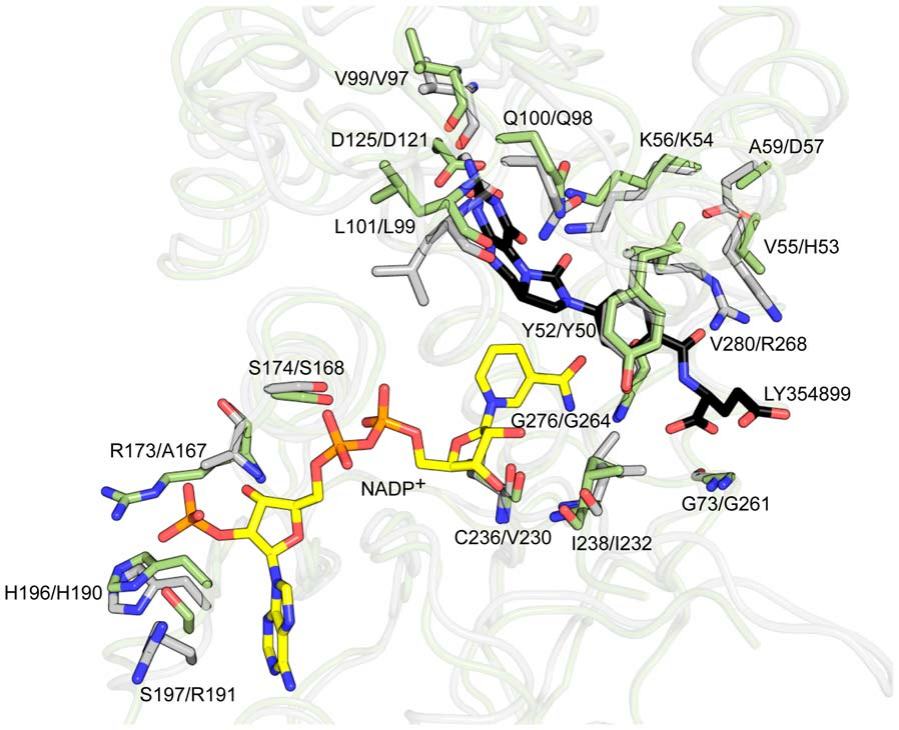
Analysis of active site residues. Superposition of the *Hs*DHCH (green) - NADP^+^ (yellow) - LY354899 (black) complex (PDB code: 1DIB) onto the remodeled *Pa*FolD structure (grey). Residues that interact with either NADP^+^ or LY354899 molecules in *Hs*DHCH and their counterparts in *Pa*FolD structure are shown as sticks.

Since the bifunctional enzyme activity of FolD is present in the pathogen of interest and in humans it may become an important selection criteria that inhibitors display specificity against *Pa*FolD over *Hs*FolD. Now, having determined the structure of *Pa*FolD we can compare the two structures and consider the likelihood of selective inhibition. The bacterial and human enzymes share 44% identity. The least-squares superposition of the *Hs*FolD structure in complex with cofactor and an inhibitor, and the newly remodelled *Pa*FolD gives an RMSD of 1.26 Å for 276 Cα atoms. The NADP^+^ binding site is mainly formed by the C-terminal domain, which displays a Rossmann-fold typical of the small dehydrogenase/reductase (SDR) family. Previously it has been noted that FolD carries an YxxxK motif commonly observed in the SDR family of enzymes and in *Pa*FolD this involves Tyr50 and Lys54. However in the SDR family, the lysine is involved in positioning the nicotinamide by virtue of binding the ribose moiety and the tyrosine provides a hydroxyl group to participate directly in catalysis [44], [45]. In FolD neither residue is in contact with the cofactor so their contribution to the enzyme activity has to be different. The tyrosine likely forms van der Waals interactions with the substrate to hold it in place and we note the hydroxyl group, positioned to bind solvent, could, in an alternative rotamer, approach the site where the cyclohydrolase reaction occurs [23]. A role for the lysine is to act as a general acid base during the cyclohydrolase reaction [46]. Two other residues are of note, Gln98 and Asp121, which are conserved in *Hs*FolD as Gln100 and Asp125 respectively. Roles for this pair of residues have been assigned on the basis of structural and mutagenesis studies [46]. The glutamine helps to position the side chain of the catalytic lysine in the active site and the aspartate interacts with and positions the pterin head group of the substrate (Figure 4).

A number of interactions serve to position the flexible 231–243 loop in the active site. These include a salt bridge formed between Asp121 and Arg234. Complementary stabilizing associations involve van der Waals interactions between the side chains of Tyr50 and Gln235 together with a hydrogen bond donated from Arg268 NH1 to the carbonyl group of Gln235. These associations would not be formed in the presence of substrate, which would directly interact with Asp121 and Tyr50.

The residues identified as being important for catalytic function are strictly conserved in FolD orthologues. We note however three non-conservative substitutions in the active site of *Pa*FolD compared to *Hs*FolD. The residues involved are His53, Asp57 and Arg268 in *Pa*FolD, which in *Hs*FolD correspond to Val55, Ala59 and Val280 respectively. The side chain of Arg268 is held in position by a hydrogen bonding interaction formed with Asp57 OD2. Alongside Arg268 is His53. The incorporation of two basic side chains in place of valines is a significant difference in the chemical characteristics at one side of the active site of *Pa*FolD compared to *Hs*FolD. This is noteworthy since it is the type of difference that might be exploited to engender selectivity into inhibitors.

### Compound screening

In advance of performing a large-scale HTS, *Pa*FolD was assayed combining DSF with the Prestwick Chemical Library of drug-like molecules (1,200 compounds) and a bespoke Maybridge RO3 fragment library of 1,000 compounds. Compounds were screened at concentrations of 1 mM (Maybridge) or between 2–8 mM (Prestwick) against 4 µM *Pa*FolD with NADP^+^ at a saturating concentration (4 mM) to block the cofactor-binding site. The assays were run at a single point aiming to identify compounds that stabilize the protein. Two standards were included on each plate, namely the inhibitors 5,6,7,8-tetrahydro-*N* ^5^,*N* ^10^-carbonylfolic acid (LY354899) and (2*R*)-2-[(4-{[(2,5-diamino-6-hydroxypyrimidin-4-yl)carbamoyl]amino}phenyl)formamido] pentanedioic acid (LY374571), at 250 µM concentration [21], [23]. The standards gave thermal shifts of +9 and +15°C respectively (Data not shown). A number of compounds in the Prestwick library gave apparent thermal shifts of approximately +10°C, however all of these were rejected due to their intrinsic fluorescence. None of the remaining compounds gave a shift greater than +1.5°C. Our experience with DSF is that such small increases on the melting temperature are insignificant and we typically do not consider values of less than +2°C as worth follow up [34]. The lack of hits suggested that a larger, more diverse set of compounds was required and therefore that it was necessary to develop a suitable high-throughout assay.

**Figure 5:**
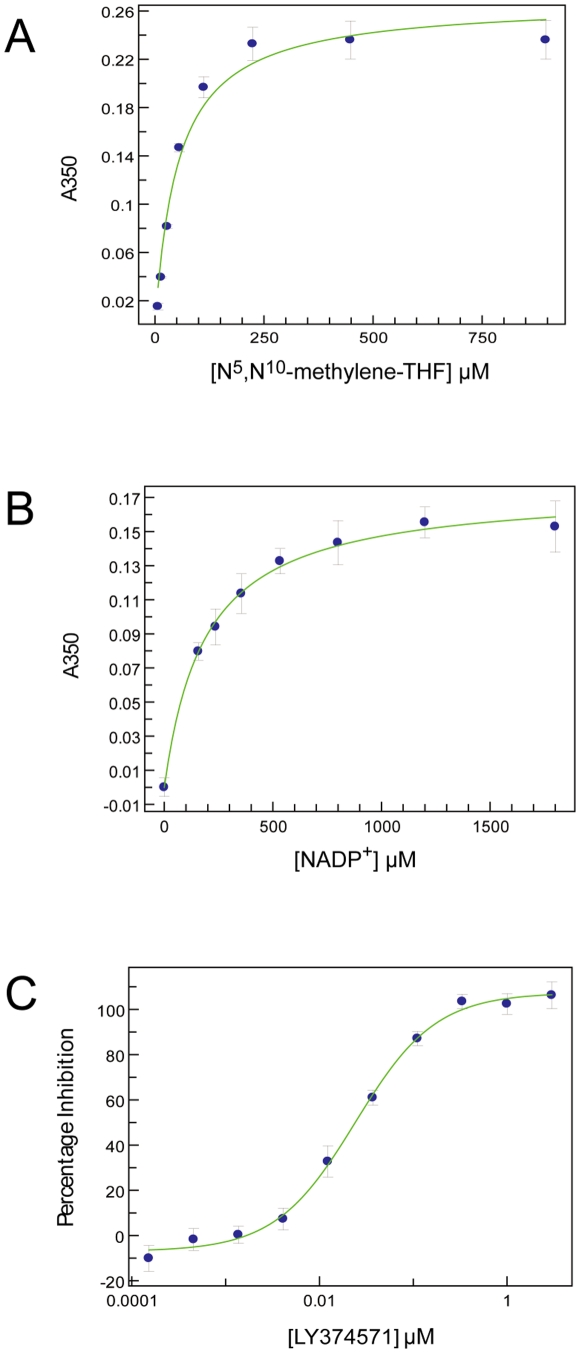
FolD assay development. (**A**) *N* ^5^,*N* ^10^-methylene-THF K_m_ determination in the presence of 1 mM NADP^+^. (**B**) NADP^+^ *K*_M_ determination in the presence of 1 mM *N* ^5^,*N* ^10^-methylene-THF. All *K*_M_ measurement data are presented as mean ± SD (n = 4) (**C**) Representative IC_50_ determination for LY374571. Data points are mean ± SD (n = 14). This representative example returns an IC_50_ for LY374571 of 27±3 nM.

A methylenetetrahydrofolate dehydrogenase assay was adapted and miniaturized to a 384-well plate format to allow HTS of 79,029 compounds [25]. Enzyme activity was assayed in an optimised buffer at 1 nM *Pa*FolD. The Michaelis constants (*K*_m_) for the substrate *N* ^5^,*N* ^10^-methylene-THF and the co-factor NADP^+^ were determined as 26±4 µM and 179±11 µM respectively (Figure 5A and 5B). When saturating concentrations of either substrate or co-factor were required *N* ^5^,*N* ^10^-methylene-THF was used at 250 µM and NADP^+^ at 1 mM. For screening the compound library *N* ^5^,*N* ^10^-methylene-THF was fixed at a concentration of 35 µM and NADP^+^ was saturating at 1 mM and the assay was stopped after 20 minutes (within the linear range of the assay). The standard compound for the screen was LY374571 which displays IC_50_ of ∼30 nM against *Pa*FolD (Figure 5C), consistent with a value of 3 nM reported against *Hs*FolD under slightly different assay conditions [23].

*Pa*FolD was screened against in-house, diverse compound libraries at 30 µM. Assay robustness and reproducibility and the overall quality of the screening were assessed by monitoring assay performance throughout the campaign. The screening data showed a mean Z′ factor of 0.79±0.04 and the mean percent coefficient of variation was 1.42±0.67 [47].

**Figure 6:**
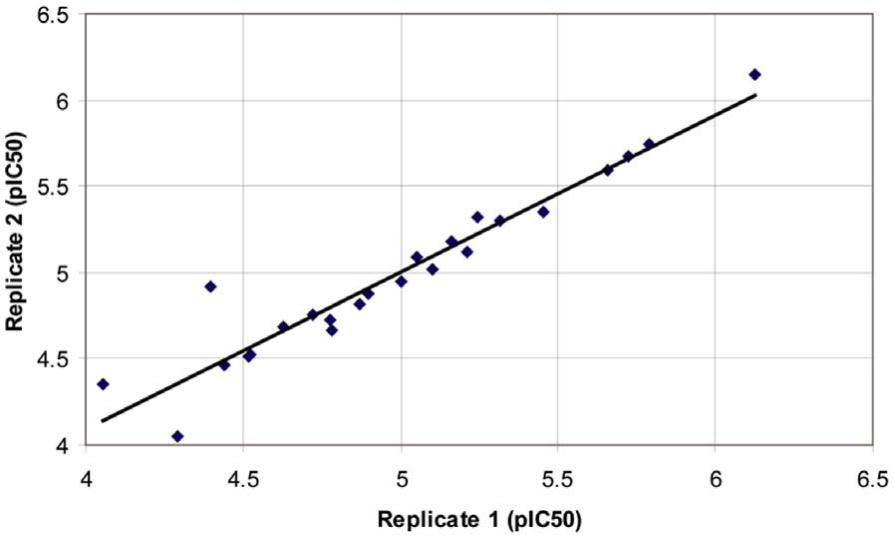
Replicate testing. Correlation between replicate pIC_50_ values for each of the 24 compounds advanced to potency testing. Linear regression of these data returned a correlation coefficient of 0.92.

**Figure 7:**
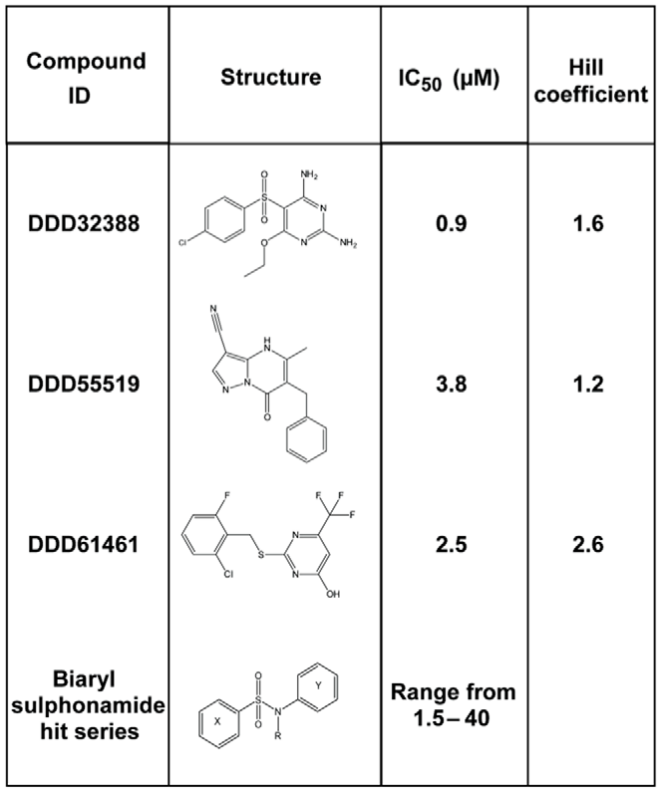
Confirmed hit compounds from hit discovery campaign. Summary of the compounds and series identified through the HTS and their respective potencies and Hill slope values.

The primary screen revealed a large number of false positive hits, which necessitated an arbitrary cut-off at 80% inhibition for retesting in duplicate. Following the retest screen and the exclusion of compounds identified as potential non-specific inhibitors of *Pa*FolD, twenty-four active compounds were progressed for potency testing. For these compounds, duplicate ten-point dose response curves were generated (ranging from 30 µM to 1 nM). We observed an excellent correlation between the two replicate pIC_50_ determinations, with the linear regression of these data returning a correlation coefficient of 0.92 (Figure 6). For each compound, residual material from the potency compound plates was subjected to LC-MS (liquid chromatography–mass spectrometry) analysis to confirm the molecular structures and sample purity. Thirteen of these twenty-four compounds were reconfirmed as active inhibitors of FolD using resupplied material, two of which proved to be unstable and were therefore excluded from the study. The remaining eleven compounds were assessed based on their core chemical structure, with eight of the compounds being assigned to one hit series of biaryl sulphonamides, and the remaining three representing unrelated singletons (Figure 7). The three singletons, DDD32388, DDD55519 and DDD61461, along with three representatives from the hit series were analysed by SPR to investigate binding to *Pa*FolD using an alternative platform (data not shown). The binding of DDD32388 and compounds from the biaryl sulphonamide hit series was weak and in the case of at least one of the sulphonamide series, non-specific. Binding of both DDD55519 and DDD61461 to *Pa*FolD was confirmed with both compounds exhibiting slow rates of association suggestive of binding at an allosteric site. The LY374571 inhibitor, a compound that binds within the FolD active site, could not compete off this binding. These results are consistent with the Hill coefficient value of 2.6 obtained for compound DDD61461 (Figure 7) since positive cooperativity induced by allosteric inhibition within the *Pa*FolD dimer would result in a coefficient greater than unity. However, the Hill coefficient of 1.2 for DDD55519 is suggestive of a non-cooperative interaction.

**Figure 8:**
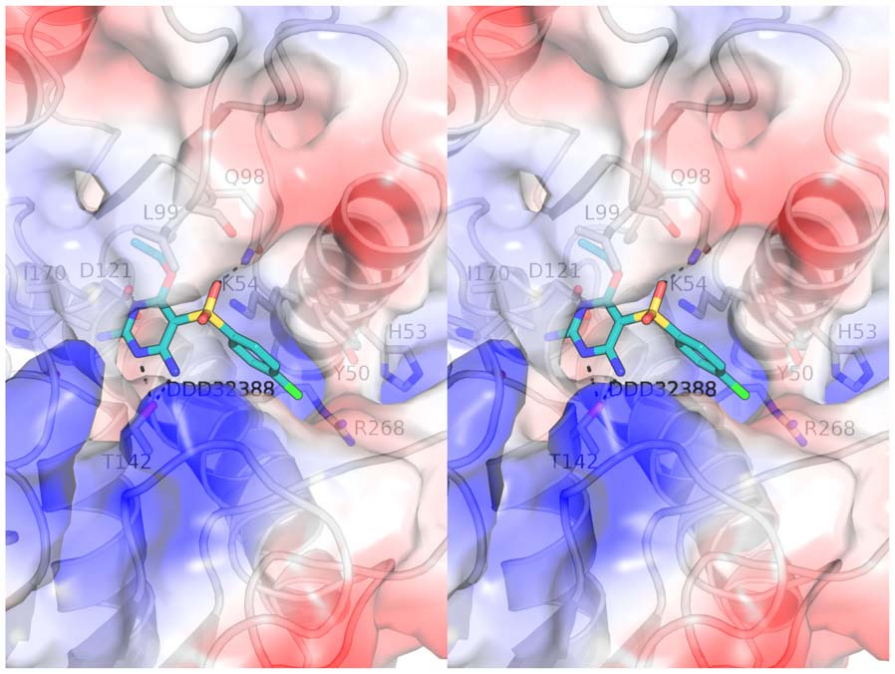
Ligand docking. Stereoview of the docking of DDD32388 (cyan) into the active site of *Pa*FolD. Potentially important residues are highlighted as sticks and with hydrogen bonds shown as dashes.

Having identified DDD32388 and members of the biaryl sulphonamide as *Pa*FolD inhibitors, attempts were made to obtain crystal structures of the complexes. In this we were unsuccessful and so used computational methods to dock the compounds into the active site of *Pa*FolD. Previous structural analyses of the FolD active site indicate no gross domain movements associated with ligand binding [26], [27]. There is however a closing down of the active site when NADP^+^ is present and from our study we observe placement of the 231–243 loop into the substrate-binding site. We carried out docking studies with DDD32388 as a lead hit using the “open” *Pa*FolD model. The poses with the best scores for both of the biaryl sulphonamide compounds were inspected and potential interactions with the protein mapped. Compound DDD32388 is predicted to adopt a similar conformation to that seen for LY354899 with the hydrophobic benzyl group stacking against the Tyr50 side chain and the electronegative chlorine substituent faces towards, yet is just of range of the basic Arg268 and His53 (Figure 8). The sulphonamide group is predicted to interact with the side chain of Gln98, whilst the head group of the molecule is slightly tilted in comparison to LY354899 and makes numerous interactions through Thr142 and Asp121. Further structural data would be necessary to accurately determine the binding of these and other compounds in the active site.

As a follow up to the enzyme inhibition assays we tested for *in vivo* efficacy, using *P.aeruginosa* PAO1 in a disc diffusion sensitivity assay. Whilst the standard antibacterial compound used, gentamycin, cleared a radius of 13 mm, all the other compounds, including LY354899 and LY374571, failed to give any clearance (data not shown). This would suggest that, whilst such compounds are highly potent inhibitors of *Pa*FolD they are not active against the bacteria and this is likely a consequence of poor uptake.

This observation raises a serious point and presents a significant challenge to this project. In order to address issues of uptake by Gram-bacteria and to design chemical modifications that influence pharmacokinetics it would be best to have an understanding of the structure activity relationships of the new inhibitors. This lack of information, due to difficulties in obtaining structural data on *Pa*FolD with the ligands, is severely limiting. Note that the new inhibitors that have been found are much less potent than the standard compounds and so there would be a requirement to enhance binding capabilities in addition to addressing issues of bioavailability.

### Concluding remarks

We prioritized the bifunctional *Pa*FolD as a potential Gram-negative antibacterial target following consideration of genetic and metabolic data and set out to assess the potential value of this enzyme for drug discovery. The crystal structure was determined and compared with that of the human enzyme. The active site is assessed as possessing the right combination of properties in terms of size, and juxtaposition of hydrophilic and hydrophobic components to warrant being described as druggable. Moreover, structural differences between the bacterial and human enzymes suggest it may be possible to discriminate between the enzyme of pathogen and host. A compound screening campaign was carried out, following the development of the appropriate assay conditions, against *Pa*FolD. Three singleton compounds and one hit series (eight compounds) were confirmed as inhibiting FolD using repurchased material and an orthogonal screening platform (SPR) was subsequently used to confirm the binding of these compounds to *Pa*FolD. Models for several of the inhibitors binding the enzyme were constructed by computational methods. None of these hit compounds showed potencies comparable to the previously characterised folate-analogue FolD inhibitors. However, the recalcitrant nature of the enzyme to crystallize in an open form or a form suitable for the soaking with known ligands or identified fragments posed our biggest challenge. Future work will focus on identifying a surrogate FolD that will provide structural data to confirm the mode of binding of these compounds and also the current ligands. It may actually be beneficial to work with the human enzyme simply on the basis that there is precedent for getting crystal structures of enzyme-ligand complexes.

Taking the results together, it is clear that although the biological and structural data suggest *Pa*FolD is an excellent target for therapeutic intervention the screening data suggest that it is in fact a difficult target to work with. Problem areas are that the hits are of low potency and it is difficult to derive a clear structure-function relationship to support the process whereby inhibition might be improved. One strategy to address this issue could be by adopting a medicinal chemistry approach to synthesise and assay different compound series. However, we caution that known, potent FolD inhibitors have no effect when tested directly on bacteria. We would be wary of carrying out further work that drives up potency against FolD but that still leaves us with compounds that have no antibacterial activity. A striking example of how similar difficulties have compromised antibacterial drug research is given by the huge effort employed by GlaxoSmithKline between 1995 and 2001. Only sixteen of sixty-seven HTS campaigns on antibacterial targets resulted in the identification of hit compounds and only five of these hits resulted in lead compound identification [7]. Despite difficulties the development of novel antibacterial compounds remains an urgent and immediate need highlighting the importance of ongoing efforts in this challenging area of drug discovery.
